# The Utilization of Waste Scraps of Old Amalgams, Etc.

**Published:** 1885-02

**Authors:** Chas. Mayr

**Affiliations:** Springfield, Mass.


ARTICLE V.

### THE UTILIZATION OF WASTE SCRAPS OF OLD AMALGAMS AND BY PRODUCTS OF DENTAL PRACTICE.

BY PROF. CHAS. MAYR, SPRINGFIELD, MASS.

[Extract from a paper read before the Conneticut Valley Dental Society.]

During a practice of only a few years there accumulate in a dentist's office all kinds of scraps, mostly consisting of old amalgams, plugs, etc., they are a small affair in themselves, but so long as dentists pay $2.00 and over for an ounce of alloy, it is worth while to give five minutes' attention to a box of amalgams containing perhaps five ounces. If there is nothing but amalgam in the scraps, I think the most legitimate use to be made of them is to remelt them, to drive off the mercury, to file them and use them again. Many dentists use only one kind of amalgam; by properly conducting this process they may get as good amalgam as the original; the best way to proceed is about this:

Put the scraps into a Hessian crucible; add for every ounce of amalgam half an ounce of cyanide of potassium; heat at a dull red heat for a couple of hours in a good draft and pour the resulting metal into an ingot; it gives a cleaner product if during the process the crucible is covered.

This ingot may be filed and will be found to give as good amalgam as the original; it is important that the heat should not be too high, otherwise some tin will volatile, and thereby the proportion of the metal will be altered.

The cyanide of potassium is an excellent flux, because it melts at a low temperature and excludes every trace of oxygen. If the operator does not care to recover amalgam in this manner, he may extract the precious metals from the amalgam in the following manner:

The old scraps are melted, with access of air, in a strong draft at a temperature at which silver melts. Almost all the mercury is thus driven off, and a considerable amount of the tin oxydizes; the resulting mass is granulated by pouring it into water and then dissolved in nitric acid which oxydizes the tin and silver, and because commercial nitric acid almost always contains chlorine, so some of the gold; the greater part of the gold, however, remains undissolved in combination of oxide of tin. After the oxidation has ceased, a powder will be found floating in the liquid varying in color from white to violet; white, if no gold is present, and colored the more the greater the amount of gold. The turbid solution is diluted with water, say to five times the original volume, it is set aside for a couple of days, and then the almost colorless liquid on the top carefully poured off. This liquid contains usually nothing but silver and copper; to precipitate the silver it is poured into a solution of salt containing about half an ounce of salt for ounce of amalgam ; a white curdy precipitate is obtained which is chloride of silver; by gentle shaking, it can be made to cling together and to settle rapidly to the bottom ; the liquid is decanted, the precipitate washed once or twice, collected on paper, allowed to dry, mixed with about twice its bulk of black flux, which is nothing but soda and flour mixed in equal proportions. This mixture is put into a sand crucible and melted at a bright red heat. A globule of pure silver is thus obtained at the bottom of the crucible, which may then be utilized further. To recover the gold, the violet precipitate from which the first solution of silver was decanted is washed a couple of times by decantation ; it is then treated with a mixture of hydrochloric and nitric acid, about one ounce for every ten ounces of amalgam ; its color will disappear and a yellow turbid liquid result. This is diluted, allowed to settle, decanted and mixed with a solution of common sulphate of iron (copperas,) impure gold is precipitated as a bluish powder, it is collected on a filter, melted in a clay scorifier with a little saltpetre and repeatedly dissolved and precipitated,

In this way alone the gold can be completely freed from the tin; the oxide of tin obtained during this process may be dried and is the commercial putty powder.

I know that these things are simple, have been told often, and contain little new; but perhaps some one has not yet heard of them, and, if so, this will have been sufficient inducement for me to give this synopsis.—*Archives of Dentistry.*

